# Developing the Concept of Working Memory: The Role of Neuropsychology^1^

**DOI:** 10.1093/arclin/acab060

**Published:** 2021-07-23

**Authors:** Alan D Baddeley

**Affiliations:** Department of Psychology, University of York, Heslington, York YO10 5DD, UK

**Keywords:** Working memory, Phonological loop, Visuospatial sketchpad, Central executive, Amnesia, Frontal lobes

## Abstract

The evolution of the concept of a multicomponent working memory is described with particular reference to the contribution from neuropsychology. Early evidence from patients with the classic amnesic syndrome, together with others showing the opposite deficit of impaired short-term but preserved long-term memory argued strongly for a separation between long- and short-term memory systems. Simulation of the short-term deficit in healthy participants using a dual task approach suggested the need to assume a three component system serving as a multi-purpose working memory comprising an overall attentional control system, the Central Executive, aided by separable temporary buffer stores for phonological and visuospatial information. An account is then given of the way in which evidence from patients was combined with the study of healthy participants to test and expand the model over subsequent years. This led to the need to propose a fourth component, the Episodic Buffer, a system that combines information from multiple sources and makes it accessible to conscious awareness. I conclude with a brief account of how the multicomponent approach resembles and differs from that of other current models of working memory.

I am delighted to receive this award, particularly since I cannot claim to be a neuropsychologist. I am however someone who has benefited greatly during a career of over 50 years from the opportunity to work with a succession of neuropsychologists all of whom have combined their considerable clinical skills with an interest in applying developments in cognitive psychology to understanding neuropsychological deficits. I spent 30 years of my research life at the Medical Research Council Applied Psychology Unit in Cambridge, England which had the remit of combining basic and applied research (Baddeley, 2019). My neuropsychological research follows this pattern with more applied work principally involving long-term memory (LTM), much of it concerned with developing tests that apply new developments within cognitive psychology to clinical problems, a commitment that still continues (Baddeley, Hitch, & Allen, 2019, 2020; Baddeley, Atkinson, Hitch, & Allen, 2021; Laverick et al., in press), while other work was more theoretically driven, using the rich data from neuropsychological patients to test and develop cognitive theory. This has been particularly prominent in developing the multicomponent model of working memory. It is this rather than my more directly applied but more sporadic involvement with LTM that forms the rest of my presentation.

My interest in memory followed a PhD funded by the Royal Mail who were interested in optimizing the design of postal codes. This was followed by a project on evaluating the quality of telephone lines by requiring people to briefly remember messages that were presented either in quiet or in noise, the assumption being that noisy speech would be hard to store and then recall. This was linked to the discovery by my supervisor that short-term memory (STM) appeared to be acoustically based (Conrad, 1964) and that acoustic similarity between remembered letter sequences (bgvtcp versus kwrsyq) had a major effect on recall accuracy, even when the letters were presented visually (Conrad & Hull, 1964). If STM was indeed an acoustically based system the perhaps it would be very sensitive to the auditory quality of the input?

My own study therefore looked at the effect of input noise on STM but took the further steps switching from letters to words (e.g., Mat, Can, Man etc. vs. Pit, Day, Cow etc.). This allowed me to evaluate the possibility that any kind of similarity might have an equivalent effect presenting a major objection to Conrad’s argument for a purely acoustically based store. To test this also included sequences of adjectives that were either semantically similar (Huge, Big, Large etc.) or dissimilar (e.g., Deep, Old, Wet etc.). I asked my participants to remember sequences of five words and did indeed find a dramatic effect of acoustic similarity with around 80% correct for dissimilar words versus 10% for similar sequences, while similarity of meaning showed a minimal effect (75% vs. 65%, happily supporting my boss’s theory of an acoustic STM system. My second step was to show that this pattern completely reversed when list length was increase to 10 words requiring several trials to learn. This suggested the simple conclusion that while STM depended on acoustic coding, LTM was semantically based. This bought me a ticket into the hot theoretical controversy of the time, whether it was necessary to assume more than one kind of memory. In retrospect, I realize that this was the true origin of my lifetime preoccupation with working memory, the system that holds information in mind while thinking.

My introduction to neuropsychology came a few years later when Elizabeth Warrington who I had known since undergraduate days asked if I would like to work with amnesic patients. I was initially skeptical, arguing that it seemed very unlikely that they would be kind enough to have their lesions in theoretically interesting places but agreed to go along and demonstrate a technique that might have relevance to the hypothesis that the amnesic syndrome might reflect a retrieval deficit. I duly arrived and it rapidly became clear that our patient did not have a generalized retrieval deficit, so what next? I asked Elizabeth what amnesic patients were like and she said that if they were interrupted they immediately forgot. This seemed unlikely given what we thought we knew about STM, namely that STM traces gradually fade over 20 s or so (Peterson & Peterson, 1959), so we decided to send our patient for a cup of tea while we made up material and tested her. We found that she did not in fact forget immediately, but rather at the normal rate expected by the cognitive literature, despite being densely amnesic.

I was surprised by the clarity and apparent purity of the dissociation and intrigued by potential raised by neuropsychology to throw light on theory. We agreed that it would be interesting to take a range of tests from the ongoing STM controversy and apply them to the group of patients that Elizabeth had carefully selected as having a very pure LTM deficit. We did indeed find broad support for a distinction between STM, preserved in the amnesic syndrome and LTM, that was, greatly impaired (Baddeley & Warrington, 1970). This fitted neatly into the demonstration by Shallice and Warrington (1970) of the contrasting pattern of impaired STM and preserved LTM which they found in a small sample of patients with left hemisphere lesions. Their patients had digit spans of around two items, implying grossly impaired STM with apparently normal language and cognitive skills, including the capacity to transfer new information into LTM. Together, the two types of patient provided a double dissociation between classic amnesic patients with impaired LTM and but preserved STM and the newly discovered STM patients with the opposite pattern, consistent with a distinction between the two types of memory that was currently the source of considerably controversy within the mainstream cognitive literature.

## Developing the Concept of Working Memory

Throughout the next few years, while maintaining an interest in neuropsychology, my main focus of attention was on the lively controversy as to the nature and function of STM. This was a very active area with a range of new experimental paradigms being developed, often with accompanying mathematical or computational models. Much of this work was subsequently captured in what became known as the “modal model,” presented in an influential paper by Atkinson and Shiffrin (1968). This assumed that information flowed through a number of sensory memory systems, often termed iconic memory for the visual and echoic memory for the auditory, before feeding into a short-term store (STS) which then transferred information to LTM. The STS was assumed to act as working memory, being responsible not only for holding information while it is transferred to LTM, but also for performing a range of control processes including rehearsal, choice of strategy and decision as to how and when to respond. The modal model continued to feature in standard texts for many years, but by the early 1970s was already encountering problems, of which two were substantial.

The first problem involved the assumption that merely holding information in the STS was sufficient to ensure transfer to LTM; it rapidly became clear that mere time in store was relatively unimportant compared to the operations performed on the material, with deeper and more elaborate processing leading to better learning as suggested by Craik and Lockhart (1972) in their Levels of Processing hypothesis. The second problem came from the previously described discovery by Shallice and Warrington (1970) of patients who had grossly impaired STM, together with apparently normal LTM. How could information get into LTM if the STM system was impaired? Furthermore, if the short-term system functioned as a general working memory, then such patients should have extensive cognitive deficits, making everyday life problematic. This was not the case, making it unlikely that the proposed STS performed all the activities proposed by the modal model. By this time, I had moved from the APU in Cambridge to the recently founded University of Sussex, where I obtained my first research grant, to study the link between LTM and STM with Graham Hitch as postdoctoral fellow.

The study of STM did not look promising at the time. It had begun to fractionate into a range of different models many focused on specific and detailed experimental paradigms with little generality. Meanwhile the modal model itself was encountering the problems just described. An obvious next step would be to focus on the patients with impaired STM who were proving problematic for the modal model, but these were rare and not available to us. Instead we decided to turn our undergraduate participants into simulated patients by systematically loading their STM and studying the impact of this on a range of different cognitive activities. Our basic paradigm was to require participants to hear and repeat back sequences of digits varying in length from zero to eight digits while at the same time requiring them to perform tasks involving processes that might reasonably be assumed to depend on the hypothetical working memory system. We wanted findings to be general rather than specific to a particular task and opted to study three different activities, verbal reasoning, prose comprehension and word list learning. In each case, we assumed that the longer the concurrently repeated novel digit sequence the greater the load on the STS should be and the poorer the performance. Indeed, if the modal model was correct, performance should break down completely when the load approached digit span.

To our surprise this was not what we found. In the reasoning task for example, response times became systematically slower as concurrent digit load increased, but only by about 50% at the maximum load of eight digits while even more surprisingly, error rates remained constant at around 5%. Broadly similar results were found for prose comprehension and verbal learning, prompting us to revise our views of STM, abandoning the assumption of a simple unitary store and replacing it with a three component model which we termed “working memory” to emphasize the fact that we were interested in its functional importance, in what it did, rather than its simple storage capacity.

**Fig. 1:**
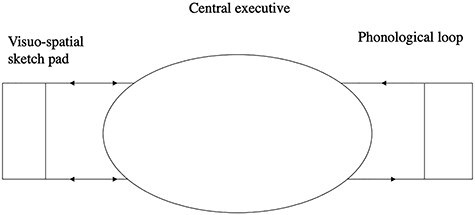
The original version of the multicomponent model proposed by Baddeley and Hitch (1974).

The three components comprised the Central Executive, an attentional control system, aided by two temporary storage systems, one verbal and subsequently named the phonological loop and its visual equivalent, the visuospatial sketchpad (see Fig. 1). Work on the model started in Sussex, continued through a very fruitful 2-year period at the University of Stirling and was finally published from the APU in Cambridge where I had returned as director, following Donald Broadbent. Our three component model first appeared as an invited chapter, an invitation we were cautious about accepting because the model was certainly not complete. Fortunately, we did accept (Baddeley & Hitch, 1974) and it has now been cited (Baddeley & Hitch, 1974) more than 17,000 times. It is still incomplete! Indeed, I regard one of its strengths is that of providing a broad theoretical framework whose incompleteness invites more detailed development while at the same time encouraging its application to an increasingly wide range of related issues both basic and applied. Such extensions are hopefully capable of increasing its scope while also potentially highlighting the limitations of the framework and driving it to evolve by stimulating fruitful further questions.

One of the most productive of such links has been with neuropsychology. As already discussed, evidence from the amnesic syndrome played an important part in separating the long- and short-term aspects of memory while the identification and study of patients with STM deficits by Shallice and Warrington (1970) highlighted the crucial limitation of the Atkinson and Shiffrin (1968) modal model. In what follows, I will give a brief account of the way in which the multicomponent model of working memory has developed, using the broad framework to highlight the particular contributions made by neuropsychology to my own theoretical development before concluding with some thoughts on the various ways in which neuropsychology and cognitive psychology are able to interact so fruitfully.

Although our model had three components our initial work focused principally on the verbal subsystem which we judged to be the easiest starting point, partly because much of the earlier literature on verbal STM was directly relevant, including my earlier work on acoustic and semantic coding. We proposed a simple model of this component which we initially termed the *articulatory loop*. This comprised an acoustic-verbal temporary store in which information would fade unless refreshed by vocal or sub-vocal rehearsal. Our earlier work on the role of acoustic and semantic similarity implied some form of language-related short-term storage system while evidence for the importance of rehearsal came from our demonstration of the word length effect, the observation that verbal memory span is strongly dependent on the spoken length of the words to be recalled (Baddeley, Thomson, & Buchanan, 1975). Thus when asked to immediately recall sequences of five words, participants recalled about 90% when these were monosyllables (e.g., stick) but only about 50% when the words comprised five syllables (e.g., refrigerator). Furthermore, the relationship between word length and performance breaks down when articulation is disrupted by the concurrent requirement to repeatedly utter even a single sound such as the word “the,” termed articulatory suppression. Such suppression of rehearsal also eliminates the phonological similarity effect, but only when presentation is visual. We interpret this as showing a direct route from audition to the acoustically based storage system, allowing the sound characteristics of the input to be registered, though not actively rehearsed, implying that with visual presentation, articulation is necessary to convert the stimulus from a visual to an acoustic code.

To summarize, our simple loop model assumed a memory trace registered in an acoustic or phonological store that fades unless refreshed. The trace can be set up either directly through auditory presentation or indirectly through subvocal articulation. This subsystem is controlled by the central executive for which it provides a relatively attentionally undemanding source of sequential verbal information which can then be strategically employed to help perform other demanding tasks. Each of the components of the phonological loop model can be studied relatively independently either by manipulating the acoustic or visuospatial similarity within the remembered material, by controlling rehearsal through articulatory suppression and in the case of the central executive, through the use of attention-demanding concurrent tasks. The strategy of using concurrent tasks together with the manipulation of similarity has continued to prove fruitful throughout the subsequent 40 years of studying working memory (see Baddeley & Hitch, 2019 for an overview).

A valuable opportunity to study the nature of the link between perception, articulation and memory came from the study of anarthric patients (Baddeley & Wilson, 1985) where a patient suffering from locked-in syndrome with a total inability to articulate, had a relatively normal memory span and showed the classic pattern of phonological similarity and word length effects. This was also the case for other patients with less drastic problems with overt articulation. (Baddeley & Wilson*,* 1985). A contrasting pattern was found in a patient suffering from word deafness who showed impaired immediate memory that was essentially due to phonological processing problem (Baddeley & Wilson, 1993). Furthermore, while patients with a pure deficit in phonological STM are rare, as Vallar, Corno, and Basso (1992) report, impairments of the phonological loop are far from uncommon in patients with a more complex pattern of aphasia. Finally, the basic phonological loop model can be linked to both its probable anatomical representation and its neuropsychological application (Vallar & Papagno, 2002).

By this point, we felt we had a reasonably good basic model of the phonological loop but were left with a rather important question. What was its function? One way of answering this would be to locate a patient with a very pure verbal STM deficit and establish first, whether a detailed assessment of her deficit was consistent with that predicted by the phonological loop concept. If so, we could ask what activities gave her difficulty. These should then throw light on the basic functions of the phonological loop.

The opportunity to study a patient with a clear and apparently isolated deficit in verbal STM occurred through a collaboration with Giuseppe Vallar in Milan, one of a group of neurology-based neuropsychologists developed by Ennio De Renzi in Milan, who subsequently proved to be invaluable colleagues. We were able to show that patient PV who had suffered a left hemisphere stroke showed a pattern of impairment that closely resembled that expected by the model, namely a very pure deficit on tests of STM such as digit span and the recency effect in free recall, coupled with normal performance on measures both of broad cognitive function and of LTM (Vallar & Baddeley, 1984a). PV’s immediate serial memory for consonant sequences showed a clear phonological similarity effect with auditory presentation, but no effect when presentation was visual, suggesting a failure or reluctance to convert the visual input into an auditory representation. Consistent with this, she showed no effect of articulatory suppression and no word length effect, again suggesting that she was not using the system in the normal way, we suspect because this would simply be feeding information into her defective phonological storage system. This was not due to her inability to articulate rapidly as her speed of speaking the number sequence 1–10 and reciting the alphabet was comparable with control participants (Vallar & Baddeley, 1984a). Subsequent research showed that, contrary to the claims of Allport (1984) that STM patients have a subtle perceptual problem, she proved to be well within the normal range on phonological discrimination, the assignment of stress to written words and judgment of rhyme, while her comprehension of individual words and short sentences was also unaffected (Vallar & Baddeley, 1984b).

Our initial assumption was that the phonological loop played an important role in language processing but this was not supported for spoken or written sentences (Vallar & Baddeley, 1987). So what was the function of the loop, if any? Could the loop perhaps be important in acquiring language in the first place? We set out to test this asking her to learn an eight word vocabulary of an unfamiliar language, Russian (e.g., flower – svieti), comparing this with her capacity to learn to associate pairs of words in her native language (e.g., castle – table), a task that we know to be based principally on semantic coding. When compared to a matched control group, PV learned the pairs in her native language at a normal rate while completely failing to master any of a set of eight Italian-Russian pairs by the time all of the controls had learnt the whole set (Baddeley, Papagno, & Vallar, 1988). This provided clear support to the hypothesis of the phonological loop as a language learning device.

In the absence of a second patient, we were unable to replicate directly, moving instead to an approach based on disrupting the phonological loop in healthy participants. We did this initially by using articulatory suppression, finding that this did indeed differentially impair acquisition of Finnish vocabulary items in a sample of English speaking participants (Papagno, Valentine, & Baddeley, 1991) while Papagno and Vallar (1992) showed that, as predicted, foreign language acquisition was differentially affected by both word length and phonological similarity within the novel word set.

This raised the question of whether a phonological loop limitation might lead to slower development of vocabulary in children. Susan Gathercole and I were able to test a group of 8-year-old children diagnosed with specific language impairment (SLI) who had the language development of 6-year-olds, together with normal nonverbal cognition. A preliminary study using existing tests indicated a particular problem in repeating back unfamiliar spoken non words, a deficit that was not due to problems in hearing or articulation. We went on to develop a test (Gathercole & Baddeley, 1996) in which the child hears and repeats non words increasing in length from two syllables (e.g., ballop) to five (e.g., altupatory). We compared our SLI children with two control groups, one comprised 8-year-olds with normal levels of both verbal and nonverbal development while the second group comprised 6-year-olds who were matched with the 8 year-old SLI children on language level. The SLI group showed a marked decline in performance as number of syllables increased, an effect that was substantially greater than that shown in either of the control groups. (Gathercole & Baddeley, 1990).

**Fig. 2:**
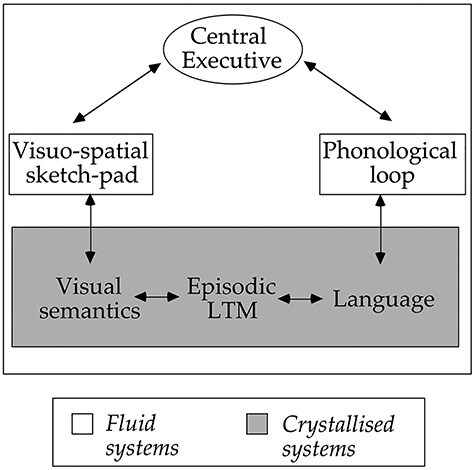
Evidence for the role of the phonological loop in long-term language acquisition leads to specifying a two way link between working memory and LTM.

A further series of studies was concerned with the link between the phonological loop and vocabulary development in healthy children. Here we found a robust association between our non-word repetition test and vocabulary, tested by speaking a word and requiring the child to select the correct one of four pictured items. A longitudinal study suggested that this was driven initially by phonological loop capacity with our non-word recognition test predicting vocabulary a year later rather than the reverse. The association between non-word repetition and vocabulary is not limited to the initial years but as vocabulary develops the association becomes reciprocal; having a good vocabulary enhances acquisition of new words (Gathercole & Baddeley, 1989a, 1989b), while eventually other factors such as executive processing and exposure to a rich verbal environment become more important. The non-word repetition test also proved to be closely predictive of problems with reading both at the individual level (Baddeley & Wilson, 1993), and more broadly (Gathercole, Pickering, Knight, & Stegmann, 2004), leading at a practical level to its extensive use in assessing reading problems in children. At a theoretical level, it fed into an overall review of the evidence suggesting the phonological loop might operate more generally as a language learning device (Baddeley, Gathercole, & Papagno, 1998) and suggesting the need to assume a direct link between temporary storage in the subsystems and long-term learning (see Fig. 2).

We proposed a visuo-spatial component to the original model, on the basis of earlier research where we had found a distinction between visual STM and LTM in amnesic patients resembling its verbal equivalent (Warrington & Baddeley, 1974), and in the case of visual STM had developed a measure of STM for complex patterns (Phillips & Baddeley, 1971). This was later developed and published as the visual patterns test, a clinical measure that was shown to distinguish between the visual and spatial components of working memory (Della Sala, Gray, Baddeley, Allamano, and Wilson (1999).

However, my first attempt to explore the concept of a visuospatial sketchpad systematically came from a study of visual imagery that was prompted by an experience while on sabbatical leave in California. I became interested in American football and was listening to the UCLA-Stanford college game while driving along the freeway. I had formed a clear image of the field and the current play when I noticed that I was weaving from lane to lane and rapidly switched to music! On returning to the UK I set out to simulate this experience. I did not have access to a driving simulator but instead used a pursuit rotor, an ancient instrument whereby the participant attempts to keep a stylus in contact with a moving light spot. I was able to combine this with a series of tasks involving visual or verbal STM, finding that that tracking disrupted the visuo-spatial but not the verbal STM task (Baddeley, Grant, Wight, & Thomson, 1973). A later study indicated that the disruptive effect of our tracking task was essentially spatial rather than visual in nature (Baddeley & Lieberman, 1980) leading me initially to propose that the whole system was spatially based. I was however subsequently convinced by my colleague Robert Logie (1986) that the sketchpad comprises separate visual and spatial components, a position later demonstrated more thoroughly by Klauer and Zhao (2004).

My own involvement in the neuropsychological basis of the visuospatial sketchpad has however been limited to a single study of an interesting patient, LE who suffered brain damage as a result of Lupus Erythematosus. She was a talented sculptor who reported losing her capacity to visualize but retaining her spatial abilities, as shown for example by her driving herself on an unfamiliar route to our Cambridge lab. Her sculpting changed dramatically from detailed realistic representations to an entirely abstract form. This was not simply a change of style since her capacity to produce simple drawings also deteriorated substantially. A careful study of her performance on a range of STM tasks indicated a sketchpad deficit (Wilson, Baddeley, & Young, 1999) while this also proved to be the case for a patient originally described as having a deficit in face memory (see Hanley & Young, 2019 for a discussion). There are of course other cases of preserved spatial but impaired visual memory (e.g., Della Sala et al., 1999; Farah, Hammond, Levine, & Calvanio, 1988) or the reverse pattern of spatial impairment with preserved visual STM (e.g., Carlesimo, Perri, Turriziani, Tomaiuolo, & Caltagirone, 2001), while as mentioned earlier, performance on the spatial Corsi blocks test dissociates from performance on the visual pattern span test (Della Sala et al., 1999).

Neuropsychological evidence played a much more important role in developing the concept of the all-important central executive. When attempting to survey our first decade of research on working memory (Baddeley, 1986), I encountered a huge gap; how should we conceptualize the central executive? Our problem was not with the common criticism that the executive was simply a convenient homunculus, a little man in the head who could be used to explain away any awkward findings. My response to this objection is that such homunculi can be useful in theorizing, as a means of acknowledging that a certain area lies beyond the scope of a developing model, given two provisos. Firstly, that one accepts that homunculus constitutes a convenient reminder of the problem area, not an explanation and secondly that the labeled area will itself subsequently need to be tackled (Attneave, 1960: Baddeley, 1998). However, it was clearly time to make a start on stage two for which I needed a suitable model of attention that I might incorporate into the existing framework.

There were several possible attentional models already in existence but unfortunately all except one were principally concerned with the attentional control of perception whereas an adequate working memory should also involve control of action. The one exception came from a technical report by Norman and Shallice (1983) and was sufficiently novel as to prove hard for them to publish in a standard journal, eventually appearing as a book chapter (Norman & Shallice, 1986). The two authors had somewhat different aims in mind. Norman was interested in explaining slips of action, ranging from everyday examples such as setting off to the supermarket on a Saturday morning and inadvertently finding yourself at your weekday office location, to other more drastic cases that could potentially result in a major catastrophe such as an air crash or a nuclear power plant disaster. Shallice’s principal interest was very different and stemmed from his desire to understand the function of the frontal lobes and to explain the sometimes bizarre patterns of behavior shown by some but not all patients with frontal lobe damage. Together they came up with a simple but powerful general model that assumes that behavior is controlled in two ways. The first assumes that many of our actions are controlled semi-automatically as a result of well-learned skills and habits. An example would be driving your car on a familiar route. You would be obeying a whole range of rules regarding action, keeping to the correct side of the road, responding automatically when the car in front brakes and not going through red lights. And yet you might arrive having little memory of any of these reliable but habitual behaviors. Suppose however that the route is blocked by an accident. A second component, the supervisory attentional system (SAS) steps in to plan an alternative route. It is this system that Shallice attributes to the frontal lobes, a system that may not be required when everything is going smoothly, but is essential when there is a need to change a pattern of action or plan future activities. This was the model I chose to incorporate as the central executive component of our model.

However, while this broad model might provide a useful distinction between two potential methods of action control it could be argued that the SAS itself is simply another homunculus. Further development is thus needed if it is to prove to be theoretically productive. Shallice chose to tackle the problem of modeling the SAS based an extensive range of neuropsychological studies of patients with frontal lobe damage, culminating in a detailed computational model (Shallice, 1988, 2002). Lacking the computational skills to adopt this method, I opted for a different approach. I proposed a list of basic capacities that were likely to be needed by any model capable of fulfilling the functions of a central executive and went on to try to study them separately. By systematically attempting to explain how the homunculus performs each of these functions I hoped that eventually with all the functions explained, the homunculus would be able to retire (Baddeley, 1996, 1998).

We began with a basic question, does the central executive function as a unitary system, or can it be fractionated into separate components? If so, what might these be? We assume that any adequate executive system would need to focus attention and to inhibit the processing of irrelevant distractors. It would need to be able to divide attention when simultaneously performing more than one activity and where necessary to switch attention between tasks. Finally it would require the capacity to access and manipulate information from LTM (Baddeley, 1996).

At this time, my active neuropsychological activities however were largely practical, concerned with the question of which standard psychological tests (if any), were able to predict memory performance in everyday life, something that our earlier research had called into doubt (Sunderland, Harris, & Baddeley, 1983). This in turn had led to collaborating with Barbara Wilson at Rivermead Rehabilitation Centre in Oxford on validating her test of everyday memory the Rivermead Behavioural Memory Test (Wilson, Cockburn, Baddeley, & Hiorns, 1989). An incidental benefit from this came through the opportunity to work with a range of potentially theoretically interesting single cases, one of whom, RJ showed the classic attentional problems associated with bilateral frontal lobe damage (Baddeley & Wilson, 1988). He was unable to achieve any categories on the Wisconsin Card Sorting Test, making 45 errors of which 43 were perseverations. His verbal fluency was grossly impaired, failing to generate any words beginning with S or V and able to generate only one animal name. This was not due to a lack of semantic information since when asked for example for an Australian animal that hops he readily came up with “kangaroo.”

His general behavior was also characteristic of the classical frontal pattern ranging from passive and apathetic to charming and amusing, while demonstrating perseverative behavior in speech, writing, and action. However, although RJ vividly illustrated the typical characteristics of the patient with frontal lobe syndrome, his problems extended beyond, including some perceptual difficulties together with dense amnesia as reflected in verbal and visual recall and recognition and his failing all 12 subtests of the Rivermead Behavioral Test. Hence, although RJ reinforced my interest in the role of the frontal lobes, the extent and complexity of RJ’s cognitive deficits limited the theoretical conclusions that could be unequivocally drawn (Baddeley & Wilson, 1988). I returned therefore to the attempt to identify and study the proposed separable components of the executive.

An obvious requirement of any plausible executive is the capacity to focus attention. While clearly of central importance to any model of attention, it was not an aspect that we ourselves explored although others, notably Cowan (2005) worked extensively on this issue. One approach is to emphasize the importance of inhibition as a means of focusing and hence maximizing the use of the available attentional capacity (Engle, Conway, Tuholski, & Shisler, 1995). Some accounts go further to suggest two modes of inhibition, one concerned with preventing intrusion from earlier memories in contrast to a second inhibitory process concerned with resisting interference from competing external perceptual or attentional demands (Miyake et al., 2000) although a recent meta-analysis has questioned the generality of this proposed distinction (Gärtner & Strobel, 2021).

In my own case, a promising line of further investigation emerged as a result of an invitation from two of our colleagues from Milan, Hans Spinnler and Sergio Della Sala, to apply the multicomponent model to the study of an extensive and well assessed group of patients suffering from Alzheimer’s Disease (ad). We decided to test the hypothesis that ad was characterized by an impairment of central executive functioning, aiming to investigate which if any of the hypothetical executive components might be compromised (Baddeley, Logie, Bressi, Della Sala, & Spinnler, 1986). We found one capacity that was particularly reduced, namely the capacity to coordinate two separate tasks. We chose two tasks that were assumed to load on different working memory subsystems, a visuo-spatial tracking task that was assumed to depend on the sketchpad and a verbal task, immediate recall of digit sequences. We tested three groups, ad patients, age matched controls and young controls. In each case, we carefully titrated level of difficulty to match performance across groups varying speed of tracking and length of concurrent digit sequence to equate performance. We then required the tracking and digit tasks to be performed simultaneously. The results were clear, with minimum disruption of performance from combination of the visual and verbal tasks for either the young or the elderly controls, together with a substantial decrement in ad, reflected in this study by an increased error rate on the verbal digit task (Baddeley, Logie, Bressi, Della Sala, & Spinnler, 1986b).

A subsequent study (Baddeley, Bressi, Della Sala, Logie, & Spinnler, 1991) monitored the performance of ad patients and controls over a year, finding much more rapid deterioration in dual task performance in the ad patients reflected in both the digit and the tracking components of the task. A subsequent study by Logie, Cocchini, Delia Sala, and Baddeley (2004) ruled out an explanation in terms of overall attentional load, finding a substantial drop in the dual task performance of ad patients, even when the two tasks were made very easy. Subsequent studies showed that this was not simply a general deficit in attentional focus since to the capacity of ad patients to sustain attention in a simple detection task proved equivalent to controls (Baddeley, Baddeley, Bucks, & Wilcock, 2001; Baddeley, Cocchini, Della Sala, Logie, & Spinnler, 1999). Subsequent research has confirmed the potential value of dual task performance as a means of early detection (Parra et al., 2009).

While we have by now accumulated extensive evidence for a deficit in dual task performance in ad patients, this does not necessarily link this deficit either to a frontal lobe location or to lack of executive control of behavior more generally. This issue was tackled by recruiting a total of 32 patients with well-established frontal lobe lesions, half of whom were independently categorized as showing evidence of dysfunctional behavioral problems while half did not show such disturbance. All were tested on dual task performance and on two standard frontal lobe performance measures, the Wisconsin Card Sorting Task (WCST) and verbal fluency and were independently rated on a range of carefully specified markers of dysexecutive behavior. When the two groups were compared, the group with behavioral problems was significantly more impaired on dual task performance, while no difference emerged between groups in their impaired fluency or WCST performance. Evidence from an association between dual task performance and dysfunctional behavior was reported in a more clinical context by Alderman (1996) based on an approach to rehabilitation using a token economy system. While the system worked well for most patients, a small subgroup failed to respond positively. These patients proved to be particularly impaired on dual task performance.

During our various attempts to link the frontal lobe syndrome with our working memory model, we became increasingly dissatisfied with the terminology. As Stuss and Benson point out the term “frontal lobe syndrome” is used to refer to an “amorphous group of deficits, resulting from diverse etiologies, different locations and variable extents of abnormalities” (Stuss & Benson, 1984, p. 3). Barbara Wilson and I suggested that a behavioral description would be more appropriate, leaving open the question of anatomical localization; instead we proposed the term “dysexecutive syndrome.” We attempted to publish a brief case for the use of this term. Unfortunately our paper was turned down by several journals where in each case, referees were evenly split between those who claimed that the distinction was obvious and those who claimed that it was obviously wrong! We eventually smuggled it into the literature via the title of the paper describing the previously described patient RJ (Baddeley & Wilson, 1988), later following up with its incorporation in an invited chapter on fractionating the central executive (Baddeley, 2002) published as part of symposium on the frontal lobes (Stuss & Knight, 2002). A recent googling of the term dysexecutive syndrome suggests that our strategy seems to have worked!

To return to our aim of fractionating the central executive, our hypothesis of a specific capacity to divide attention between two or more tasks, seemed to have proved fruitful, at least as applied to Alzheimer’s Disease. Our second proposal, the capacity to switch attention between tasks however proved less promising. Although the study of attention switching was moving from a neglected to a highly active topic, our own attempt to link it to the central executive was not strongly supported. One study based on a traditional switching paradigm, suggesting a surprisingly substantial contribution from the phonological loop rather than the executive both over the short-term (Baddeley, Chincotta, & Adlam, 2001) and even more surprisingly in a relatively long-term paradigm (Saeki, Baddeley, Hitch, & Saito, 2013). More generally, despite its early promise, (Allport, Styles, & Hsieh, 1994; Rogers & Monsell, 1995), the field appears to have evolved into a range of relatively narrow paradigms associated with micro models. At present, it seems likely that there may be several ways of switching tasks, rather than a single executive capacity.

Up to this point therefore, despite their potential importance, neither attentional focus nor task switching has played an important role in developing the multicomponent model although if established, neither capacity in principle presents a major problem for an expanded version of the model. That was not the case for the fourth capacity proposed for the executive, namely that of providing an active interface with LTM. This problem was brought to the fore by the attempt to develop a measure of individual differences in overall working memory capacity by Daneman and Carpenter (1980), two investigators interested in applying the concept of working memory to prose comprehension. They argued that the essence of working memory is the capacity to maintain information while simultaneously processing it, attempting to measure this capacity using an apparently simple task which involves reading out loud a sequence of sentences then attempting to recall the final word of each. Performance proved to correlate highly with the comprehension score of on the Graduate Record Examination, and subsequently with a wide range of tasks that could reasonably be assumed to depend on working memory capacity. These extended from prose comprehension to reasoning and from eye-movement control to fluid intelligence (Engle, 2002; Engle & Kane, 2004). While I was gratified that working memory was proving to be so important both theoretically and practically, there was a problem. Was the three component model capable of explaining performance on the working memory span task itself? The central executive was assumed to be a purely attentional system (Baddeley & Logie, 1999) while neither of the temporary storage systems had the capacity to process and hold three unrelated sentences.

We were already encountering problems in explaining the fact that immediate verbal memory for brief sentences could reflect influence of both semantic and phonological information at apparently the same time (Baddeley & Ecob, 1970) and was able reflect simultaneously the influence of both phonological and visual similarity (Logie, Della Sala, Wynn, & Baddeley, 2000) This raised the question of how the various streams of phonological, visual, and semantic information could be combined and held without a multidimensional coding system We were also puzzled by data from two highly intelligent but densely amnesic patients who as expected scored zero on both the Rivermead Behavioral Memory Test and on the delayed prose recall of the WMS. Atypically however, they performed well on immediate recall of the short story component of the Rivermead test with scaled scores 12 and 9.5 (Baddeley & Wilson, 2002). How could our model of working memory hold an extended paragraph? It was time for a rethink.

**Fig. 3:**
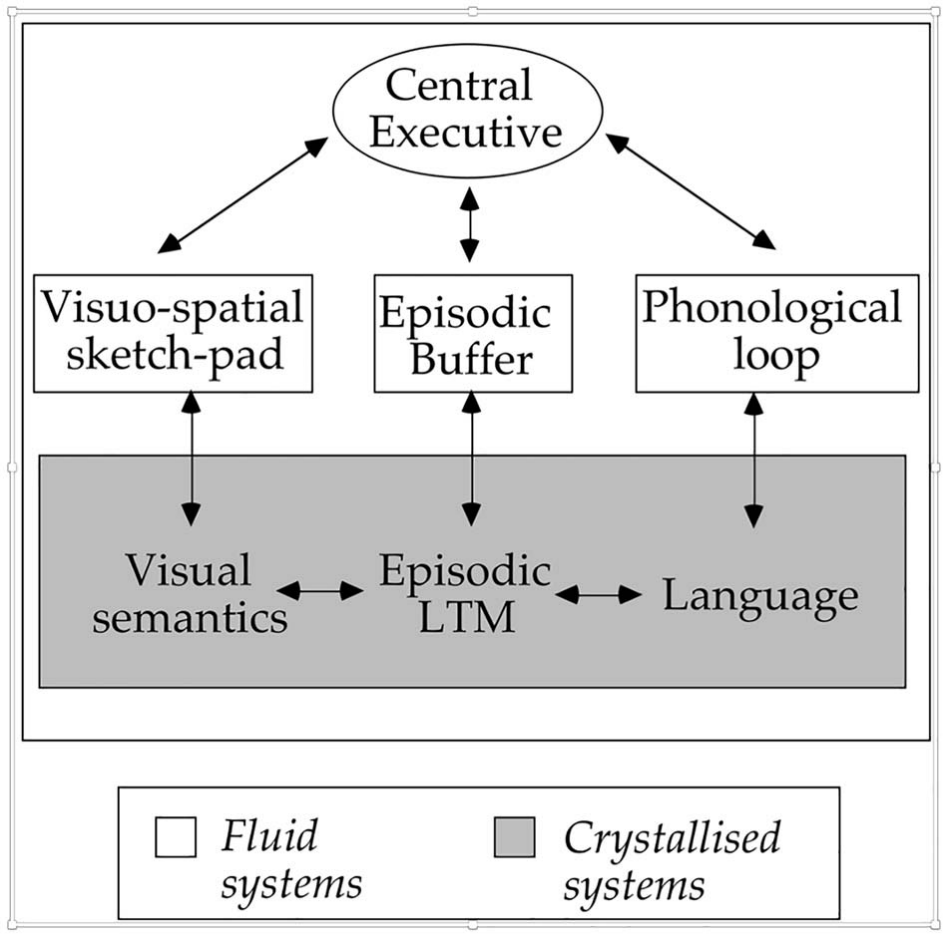
The episodic buffer is proposed as a multi-code storage system that links the components of working memory with both LTM and conscious awareness.

The result was the proposal of a fourth component to our model, the “Episodic Buffer” (Baddeley, 2000). This was assumed to be a temporary storage system that was capable of maintaining information based on a range of different codes, bound together into integrated episodes (see Fig. 3). We assumed that such coded information could arrive either directly through perception, through the sketchpad or loop, or indirectly from LTM. I made the important further assumption that the contents of the buffer were accessible to conscious awareness. A final tentative proposal was that access to the buffer was dependent entirely on the central executive. A significant advantage of our new concept was that it formed a bridge between our own approach and that of US colleagues notably Cowan’s (1988) embedded-processes model which assumes that working memory reflects the focus of attention on an activated portion of LTM. The capacity to focus attention in Cowan’s model is broadly equivalent to the central executive while the activated portion of LTM to the episodic buffer. In short, despite their apparent differences the two models approach the question of attentional control in broadly similar ways (see relevant chapters in Logie, Camos, & Cowan, 2020 for further discussion).

Much of our work over the last decade has been concerned with attempting to demonstrate that the concept of an episodic buffer can indeed be fruitful in generating tractable questions that allow the model to be developed further. For example, we began as mentioned earlier, with the assumption that the episodic buffer plays an active and essential role in binding features together into episodes and that information for this is provided via the central executive. By systematically blocking the three components of the model using dual task methods, we were however able to show that this was incorrect in two ways. First, the central executive is not essential for providing access and secondly the process of binding typically occurs at points before accessing the buffer. In the case of visual binding, it appears to occur during the later stages of visual processing while in the case of language, it typically relies on linguistic processes in LTM. These modifications in turn led us to an area we had previously neglected, namely a more detailed analysis of the attentional capacities of the central executive. The resulting evidence drove us to propose a single executive system of limited attentional capacity which could however be divided and biased to focus principally on either perceptual input or executive processing (Hitch, Allen, & Baddeley, 2020), a proposal that fits neatly with conclusions from colleagues approaching the issue from the viewpoint of visual attention (Chun, Golomb, & Turk-Browne, 2011).

**Fig. 4:**
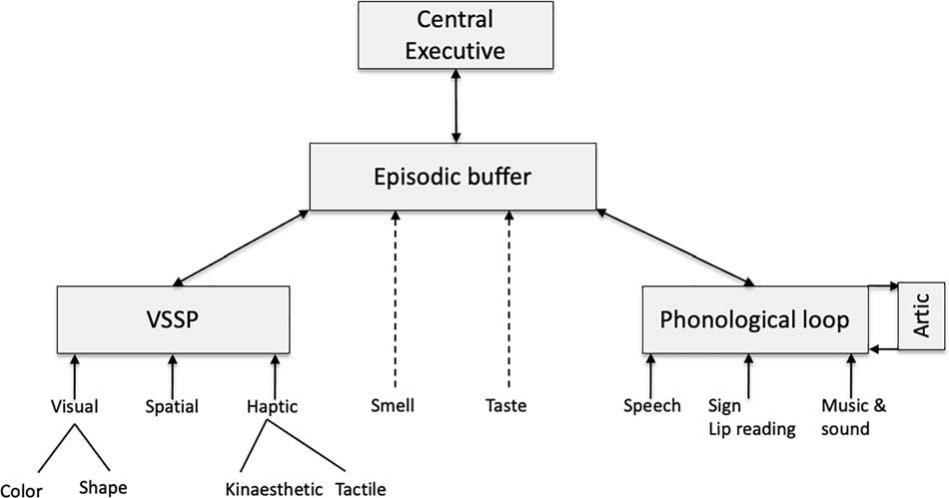
The current model illustrating the flow of information from perception to the episodic buffer.

Our current model is summarized in Figure 4, which emphasizes the various sources of input from perception to working memory (Baddeley et al., 2019, 2020). Two broad streams of information are proposed, each coming together in the visuospatial or the phonological subsystem. These allow the combination of a range of inputs, in the case of the phonological loop, information from sound together with linguistic information presented auditorily, visually via printed text, or through in sign language or lip reading (Wilson & Emmorey, 1998; Rudner & Rönnberg, 2008). In the case of the sketchpad, the confluence allows visual information on color shape and location to be spatially combined and linked to information based on touch which itself represents input from a number of kinaesthetic and tactile receptors. Both of these streams are potentially combined in the episodic buffer and hence made available to conscious awareness. As the dotted lines in Figure 4 indicate, we assume that smell and taste also have access to the buffer but have not so far investigated this. Finally our model will at some point need further specification the way in which it controls of action if it is not to be locked into permanent rumination. There is clearly much scope for further development which I am sure will continue to be enriched by input from neuropsychology.

This leads on to my final reflections on why neuropsychology has proved so fruitful in developing the multicomponent model of working memory. Part of the answer is that the level of explanation between neuropsychology and the rapidly developing area of cognitive psychology during this period was close enough for clinicians and mainstream cognitive psychologists to build common theories, even in areas as complex as working memory. I think it has proved much harder to link the complexities of working memory with methods available to neuroimaging over this period, although hopefully, this is beginning to change. In my own case at least, the link with neuropsychology has been made possible by the opportunity to work with active clinicians who regularly test patients and who in addition to their clinical skills have the expertise to identify patients of potential theoretical significance and a willingness and ability to find time to collaborate with less clinically sophisticated experimentalists such as myself. In this respect, I am particularly indebted to Elizabeth Warrington, Barbara Wilson, Sergio Della Sala, Beppe Vallar and more recently Faraneh Vargha-Khadem. Much, though by no means all of the neuropsychological contribution to the development of the multicomponent model of working memory has come from single case studies. This can be seen as a questionable source of evidence, and is probably less widely accepted in North America than Europe where the single case tradition is more established. Recent examples of such doubts are provided by Morey (2018a, 2018b) and Morey, Rhodes and Cowan (2019)) although unfortunately, such criticism can reflect apparent difficulties in adequately understanding and reporting some of the relevant data and procedures (see comments by Logie, 2019: Hanley & Young, 2019, and Shallice & Papagno, 2019). To some extent, this reluctance to accept single case data is understandable as patients with very specific deficits in capacities of theoretical importance are rare and it is neither ethical nor practical to make such patients widely accessible. It is therefore particularly important to replicate, preferably using the method of converging operations within each study ensuring that conclusions are based on a range of different modes of testing, to carefully rule out alternative explanations, hopefully followed by replications from other laboratories before drawing strong theoretical conclusions (Shallice & Papagno, 2019). Such theoretical developments should then, in principle, be more widely clinically applicable across patients whose deficits are complex but no less important. Much of my applied work in neuropsychology has focused on attempting to use developments in cognitive psychology to the understanding and assessment of memory problems in neuropsychological patients, some based on developments in our understanding of working memory, but probably more in attempts to deal with problems of LTM.

But that is another story (Baddeley, 2019).
